# Supplementary material for: Exploring HIV risk perception mechanisms among youth in a test-and-treat trial in Kenya and Uganda
Source: PLOS Glob Public Health. 2024 May 2;4(5):e0002922. doi: 10.1371/journal.pgph.0002922 (PMC11065277; doi:10.1371/journal.pgph.0002922)
Supplement: S1 File — (DOCX) [file pgph.0002922.s001.docx]

**SEARCH Qualitative Code List (15 May 2014)**

*IDI:*

*Aim - Characterize social norms, beliefs and practices related to HIV testing and disclosure; HIV-related stigma; sexual risk behaviors, and use of ART and other services*

- *What are the baseline attitudes, beliefs and social norms surrounding HIV testing, disclosure, and ART among community members?*

|  | **Code name** | **Code definition** | **Example** |
| --- | --- | --- | --- |
| Demographic characteristics | | | |
| 1 | Marital status | Any description of the participant’s marital status, whether or not the person is married. | **I: For how long have you lived in this village?**  P: I have lived for long. I got married here in 1977. |
| 2 | Place of residence | Any description of how long someone has stayed in the community, whether they consider this community to be their home, where their usual place of residence is, etc. | **I: Do you consider this community to be your home?**  P: Yes I can say so because I have lived here and it is my dwelling place now. |
| 3 | Place of birth | Any discussion on the participant’s birthplace. | **I: Were you born here?**  P: I was born in Sakwa. |
| Community characteristics | | | |
| 4 | Community context | Any description of the community that is not about problems/issues, how they came to live in that community, what people are doing for etc. | People cultivate bananas mostly, they also plant beans and cassava and cattle keeping. |
| 5 | Political history | Any description of political unrest, issues such as “camps”, poisoning of community members, history of rebel activity and social unrest. | I: Other than the limited drug supplies, home based HIV tests and other descriptions, how else can you describe Kameke community?  P: some silence. (Facial expressions at this point seemed to hide sensitive information)  I: I mean the peoples’ beliefs, behavior, health and any other description of this community.  P: Way back some people went to the bush (meaning had become rebels) but now that is no more people are now development-oriented. At some point there was poisoning of the people, which also stopped.  I: Really?  O: Yes! The place is now peaceful and visitors can now rent in the trading centers and live peacefully. |
| 6 | Community problems | Any description of a community problem such as poverty, education, illiteracy, infrastructure, etc. excluding HIV/AIDS or other health problems. | **I: If you could change anything about this community what would it be?**  P: I would try and see how drug abuse amongst our youth can be eliminated because some of them are involved in drug abuse, which leads to non-economic growth in the community.  **I: What are some of the major problems that are faced by members of this community**?  P: Being that we stay at the border which is surrounded by many tribes, there is a lot of insecurity where people are being killed …one of the village elders was recently killed by unknown individuals. |
| 7 | Socio-economic factors | Any factors involving income, occupation, and community’s economic development thus the social standing or class of the community as it reveals inequities in access to resources, plus issues related to privilege, power and control. | **I: How do the community members cope with some of these problems?**  P: It is very strange because when it comes to drinking water, the community assumes that the lake water is clean in the morning hence, they fetch it very early in the morning for domestic use. Agriculturally, we wait for rainfall. Like now, you can see nothing is happening because there are no rains and not everyone has land that borders the lake. It means that they cannot do any irrigation; otherwise, for those who have land bordering the lake, they use it for commercial purposes.  **I:If you could change anything about this community what would it be?**  P: As a community leader, I do encourage many to participate in farming because there is no major business that somebody can engage in apart from *omena (small fish)* which does not do well because we are currently receiving less fish from the lake as compared to earlier times.  **I: Can you tell me about this community?**  P:…. Otherwise, fishing business has been infiltrated by foreigners who benefit a lot from the business unlike the locals….  **I:You have mentioned high levels of death cases and availability of the drugs in the hospital, why are many reluctant to seek medication at these hospitals?**  P:Taking drugs is not an easy task even though I have not seen these drugs, but they say that some are larger in size and the fact that they are taken daily, forces someone to take them with porridge yet food is another problem in this community. There used to be enough fish in the lake but big fish are currently exported and people are equally not giving time for the small fish to grow. We have no other business apart from fishing which is not easy as at now since we have foreigners who have invested a lot in the lake denying the locals an opportunity to do fishing business. At the end of the day people must die because it is food that support good adherence to these drugs. |
| 8 | Health problems | Any description of health problems/issues impacting health including malaria, diabetes, hypertension, TB, etc. excluding HIV/AIDS. | P: The very first one is lack of adequate and clean water. There are few bore holes here in Kameke.  I: Please go ahead.  P: Bad roads often cause maternal deaths. The health facilities are also very few and distant from many households. Doctors are also very few.  P: As a leader, I would advocate for more health workers to help in treating the patients. There is right now a very big problem of diabetes in Kameke. There is no help in terms of the machine that checks for blood sugar levels in the health centre. If given the mandate I would make sure the diabetes testing machine and medicine is availed in our health centre. |
| 9 | Response to problems | Any description of what the community has been doing to address the problems (general or health specific issues) in the community or how the community is dealing/coping with the problems. | What we do in our community to see that we fight poverty, we have formed saving groups, like us the women we have formed a group called “save and borrow”. You find that we lend each other money, put in interest, it is a group, we gain interest, in about 1 or 2 years we share that money. So when you come to know that your child has been sent away from school for school fees, you go there get money and pay for your child at school. When the time comes you share your money like the rest, you buy your goat and put it at home, you buy a nice a dress and look good among people. You also cultivate and make sure that you get money to save with this group, and when you buy that goat so that when another group starts you get money to save with the group. That is what we do; we work so hard to ensure that we come out of poverty. |
| 10 | Socio-cultural factors | Any form of cultural practices by community members | I: How are people copying with HIV as a problem in this community?  P: That is very simple. One, there are different types of people in this community; there are the well-educated, less educated and non-educated. Our Luo culture also plays a big role in the community. Let me tell you that if there is a disaster in the making, a disaster in operation, culture is a disaster. There is still wife inheritance practiced among the community members. Two, the informed people are not very ready to share the information they have with others nor empower the community on HIV/Aids. You find that a good percentage of the community members still adhere to cultural beliefs and would prefer to hear HIV talked about outside their community and by those from outside their community.  **I: How did you meet this partner?**  P: He is my brother in law and as our culture dictates, the first option as the second husband should be a brother in law.  **I: You told me earlier on that your husband passed on in 1997. You are now telling me that you have not had a partner for the past six years. Could you tell me more about it?**  P: I had one partner (second husband) after my husband’s death who assisted me to build a new home as tradition requires but we left each other thereafter.  **I: How do people feel about HIV in this community?**  P: People are aware of HIV and it is only a few who still associate it with witchcraft. There are situations where you really suspect that one is HIV+ but you will find that some people will still seek the help of traditional healers. |
| HIV/AIDS | | | |
| 11 | HIV/AIDS knowledge | Description of how the respondent became aware of HIV and general knowledge about HIV/AIDS in the community. This includes measures in which an individual or community members get aware about issues concerning HIV in general. | I: What is being done to address the high prevalence of HIV in this community?  P: We have tried to educate people especially the youth about the dangers of contracting HIV at an early age. We also educate them on how to protect their lives against the disease by testing first before having sex and even using condoms for protection.  I: Where do they get this information from?  P: There is no where we do not teach from. If someone fails to go to church, when we find him in bar we tell him. We even tell them that we have boxes in bars and lodges if you fail to buy a condom they are there for free. We run educational sessions over the radios, TVs, those who can access TV; everyday they put talk shows on HIV on radios. There is nowhere the information does not reach, in churches, in mosques and everywhere.  P: How we fight them to ensure that they are eradicated?  I: Yes.  P: So the way we fight them is that if we get a seminar from the health workers, they put us together and they teach us, they teach us that every three months we have to go and test for HIV. |
| 12 | HIV/AIDS perceptions | Description of respondent or community attitudes and perceptions about HIV/AIDS in the community, OR descriptions of community attitudes and perceptions about HIV/AIDS in the community. | P: They do not want the young generation to get HIV. The men nowadays escort their pregnant wives for HIV tests and delivery too here in Kameke. They want their unborn children to be born HIV free. They also believe that HIV affects their plans for the family. Some encourage their fellow sick people not to spread the disease. |
| 13 | HIV/AIDS morbidity | Description of HIV/AIDS related deaths, effects of HIV/AIDS morbidity on people, families, and the community. | **I: How are people in this community coping with the problem of HIV?**  P: You may find that in a particular family both the father and mother have died of HIV/Aids. So, you find that an elderly grandmother comes in to take care of the grandchildren. It is generally hard because sometimes even the children are infected. This problem is with us and it is hard to understand it. |
| 14 | HIV/AIDS severity | Any discussion about how big of a problem HIV/AIDS is/HIV prevalence rate, whether HIV/AIDS is a bigger or smaller problem than other issues in the community, the severity of HIV/AIDS in the community currently, the severity of HIV/AIDS now as compared to the past. | P: Way back there was a big problem. The moment people would get to know that person X was HIV positive people would run away from them and not share cups or plates with them. The whole community would in a short time know that person’s status. It was a serious issue I tell you (Gives posture with raised eye brows).  **You mentioned earlier that as a leader the experiences you face with HIV is stigma. Tell me, is HIV a problem in this community?**  P: Ok! You know, that question is a little bit technical but I will answer it this way; you cannot tell by looking at someone or anybody just walking around to be HIV positive but there are situations that may make you suspect a person’s condition. As a community leader, I have often wondered about the death rate among the young people which has often got me thinking of HIV presence in the community. But I can generally say that the HIV prevalence rate is high in this community. I feel deeply touched by that but, it forces me to plainly talk about it. |
| 15 | HIV/AIDS transmission | This includes participant’s or community’s opinions/views on how HIV is being spread in their community, description of what they think is contributing to HIV transmission in their community. | P: …..It might be an individual’s fault that they carelessly slept around but this does not mean that you get isolated…..  P: Yes, some bar owners bring ladies from outside this community and they do not know how they are and they bring them to attract customers. Even this redundancy leads to the youth to get HIV. The high unemployment levels especially among the youth gets them involved in drinking, playing pool and sleeping around without using condoms thus contracting HIV. |
| 16 | PLWHA_ personal | Discussions of personal perceptions (positive or negative) about how they view PLWHA/does the participant know someone who has/died of HIV/AIDS, how do they feel about these people, how would they react to someone telling them they had HIV, beliefs about PLWHA. | P: I would tell him or her to accept what the doctors will have instructed since his or her days can be increased with taking ARVs. I would also advise that person on being calm as much as possible at such a delicate time.  I: Supposing someone you know told you that he/she is infected with HIV, how would you react?  P: First of all, I could be shocked. (facial expression of fear)  I: Why? Can you explain a little?  P: Because no one loves death. But on the other hand, I will feel sympathetic and judgmental.  I: Did you say judgmental?  P: Yes.  I: Why?  P: Because some people may use it as a trick to get something from you.  I: Something, Like what?  P: It can be inform of advice or material benefits, which may not be directly related to the problems they have, but taking advantage of a situation to gain out of it. However, after I have analyzed the situation, I will be able to see how to help or not to help at all.  I: Like which help will you be willing to offer?  P: Advice on how to live positively and where to access help like TASO and AIDS Information Center. |
| 17 | PLWHA_ community | Discussions of community norms/attitudes/beliefs regarding PLWHA (can be positive or negative). | P: Thank you, I am happy with what KEMRI is doing, HIV has been a major problem and KEMRI is coming up to find out the best interventions. There is a lot of HIV related stigma and people think that once one tests positive, then it is all about promiscuity. This is why we talk to people about prevention, care and nutrition. Many are dying due to stigma and fear and this is one thing we are trying to fight very hard. |
| 18 | HIV advocacy | Description of personal experiences with speaking to people about HIV/AIDS generally, giving advice or counseling people in the community/friends/relatives.  *NOTE: THIS CODE WILL OFTEN BE DOUBLE-CODED WITH “HIV/AIDS conversation” | **I: Do you talk to people about HIV/Aids regularly?**  P: Yes, I often talk to people. Sometimes, when I go to a certain household and find a sick person and this person is still looking young, I tell them that “I can see you are not in good health and you have children to take care of, don’t you care to know your HIV status instead of waiting to die and leave the children to suffer after you are gone”. I usually don’t hide my HIV status to them especially when I want to encourage them to seek medical care. |
| 19 | HIV/AIDS conversation | The description of the last specific conversation the participant had regarding HIV/AIDS. | I: It is encouraging to hear that, can you then tell me about the last conversation you had with someone when you talked about HIV?  P: The last conversation was with my wife when we were watching T.V. Dr. Venand was talking about HIV/AIDS and said that “every person has a right to be tested including gays”.  I: When was this?  P: Last week when I was watching T.V with my wife at home.  I: What did you talk about?  P: We talked about HIV/AIDS testing and its importance in relation to proper planning.  I: Okay!  I: Why were you talking about this at that time?  P: We were contributing to the conversation on T.V which was discussing the recently passed anti- homosexuality Act.  **I: Can you tell me about the last conversation you had with someone when you talked about HIV?**  P: Even recently I talked to my step sister when she was down and looking bedridden.  **I: When was this?**  P: The week before last week.  **I: What specifically did you talk to her about?**  (*Hesitates to answer and the facilitator asks the question again*)  P: Ok, I had gone to visit with her when she was very sick. Upon inquiry, she told me that she was feeling very unwell, her whole body was aching and for sure she looked emaciated. I then requested her to seek further medical attention and encouraged her to take HIV test.  **I: Why were you talking about this at that time?**  P: She was very ill and I had suspected that she was engaging in sexual activities with the young fishermen since her husband had died. |
| 20 | HIV services | Description of availability, accessibility, and affordability of HIV services in the community. | I: In your opinion; do you see that people have the urge to get involved in activities related to HIV testing.  P: To be honest people get involved; only that in the past few days what was bothering people a lot was transport because the health facilities were so far. Everyone needed to go to Mbarara to get HIV treatment. If you are to count transport money every month, but currently at this facility of ours Nyamuyanja health center IV the services are now available for free  I: When did these services start at the H/C IV?  P: ARVs’ services, I think it is about now six months; it is not a long time. I now see people come here and they do not fear and people are beginning to change and are starting to get treatment this side because of transport which used to disturbed them a lot. And the other question that they always ask me they say that, “now the health facility has just started to give HIV treatment what if it eventually gets finished? If we swallow the drugs and they get finished, will they send us to Mbarara, yet we had failed to pay for transport?” I assured them that as long as the health facility has already started to give HIV treatment, they cannot run out of drugs, because when the other ones see that the drugs have reduced they continue supplying them. |
| 21 | HIV testing_ personal | Discussion of personal experience with testing, personal motivation to test for HIV. Includes discussions about knowledge of HIV status: avoiding knowing status, wanting to know status, feelings and beliefs about knowing one’s HIV status. | P: I would impose a rule whereby all must go for HIV testing. The fact that it is voluntary creates hesitation and many people therefore take long before going for the test. It is always my joy that I get tested many times. We used to have some Home Based HIV testing which I no longer see nowadays. When they were there, I used to instruct everyone in my household to get tested… it is good news that we have drugs nowadays where one would just continue the normal chores while on these drugs. (*Laughter*). |
| 22 | HIV testing_ community | Perceptions of community norms regarding HIV testing in the community. Includes discussions about how others feel/think about knowledge of HIV status: avoiding knowing status, wanting to know status, feelings and beliefs about knowing one’s HIV status. | **I: Do you think that the availability of drugs called antiretrovirals to treat HIV might influence whether a person is willing to test for HIV?**  P: In fact many have gone for the test because of these drugs. Some people do not want to test until they are very sick, this is when they think of ARV hence go for test.  **I: You mentioned that some people do not want to test out of fear, what brings about this fear?**  P: It is out of suspicion depending on how their movements have been in the past. Many are ready to test when they are in their sick beds.  **I: How do people feel about HIV/Aids in this community?**  P: I think many people are just looking into how they can know their HIV status and thereafter seek care. |
| 23 | HIV disclosure_ personal | Description of whether the participant has personally disclosed his/her status/test results to anyone, what made him/her decide to share his/her status/test results. | **I: Can you tell me about your experiences sharing your HIV test results with other people after you were tested?**  P: Before I tested, I used to feel guilty…that sad feeling of “*how I will leave my children behind*”. Lately, I have been saying that I will die just like anybody else *(laughter).*  **I: Have you shared your HIV test results with anyone?**  P: Yes, I have shared with my co-wife who happened to be positive too and we used to make fun that if the drugs are withdrawn then we are going to die.  **I: Tell me about your decision to share your test results.**  P: I had witnessed those who died in pathetic conditions and none was ready to nurse them. We therefore decided to share our status with others to avoid the same happening to us.  **I: Is there anyone you want to know your results that you have not told yet?**  P: None. |
| 24 | HIV disclosure_ community | Discussion of norms around disclosing one’s status/test results with other, motivation to do so or barriers not to. | I: We are still continuing please do not get tired. Why do some people fear to disclose their HIV status to those whom they are close to, their partners, children or others in the community?  P: Some people fear to be segregated.  I: Apart from fear for segregation, is there anything which may affect disclosure of ones status to his close relatives?  P: Fear of being segregated is the major factor that I would like you to take note of but I can also talk about fear for missing opportunities in future if people learnt that you are HIV/AIDS Positive.  I: Okay!  I: Come on, can you share with me some of the opportunities they fear to lose?  P: Like political and leadership opportunities in the community. People will not be willing to give someone responsibility who is going to die soon.  P: No…a particular woman in a certain congregation openly asked for water to take her drugs which was faced by mixed reactions from the congregation but she was bold with her HIV status and she became my role model and inwardly I thought that people need to be like her. |
| 25 | Antiretrovirals_personal | Discussion of how the participant came to know about antiretrovirals, whether the participant is familiar with the drugs, knowledge or concerns about the drug - use, cost, availability, etc. | I: There are drugs called ARVs which are used to reduce the HIV viral load and growth. Have you ever heard about them?  P: Yes, I have heard about only their name but I have not seen them physically. I only know about septrin.  I: Ooooooh! What have you heard about ARVS?  P: What I know is that the HIV infected people take those drugs.  I: Yes you are right HIV infected people take ARVs. What else?  P: Those drugs are distributed to the patients in the health facility by the doctors.  I: How did you hear about ARVs?  P: I heard ARVs from the outreaches and radio talk shows. |
| 26 | Antiretrovirals_community | Discussion of community perceptions around antiretrovirals, what people in the community think about the drug – use, cost, availability,etc. | I: You are right the organization that used to give out those food staffs stopped. Otherwise what do people in Kameke talk about ARVs?  P: They say that" so and so used to be thin and about to die but the ARVs have helped him or her in looking good and healthy".  I: That is amazing! I would like to know how people react to the HIV infected people in relation to the availability of ARVs.  P: The way I see, people look happy about ARVs and come for the HIV tests freely with the intension of knowing their status so that they could start on ARVs if found positive (He did not seem to understand the question).  I: Thank you for that. Just take an example that I am HIV positive and come from Kameke, how would people look at me? Compare two situations when there were no ARVs in place and now when ARVs are available in the health facilities.  P: In Nyakoi trading centre last month, I heard people saying that the HIV positive people are nowadays looking good and healthy as compared to the time when there were no ARVs. They added that before ARVs were brought, people used to look so bad with their thin bodies and rashes all over. |
| 27 | HIV care | Description of whether or not the participant, if HIV-positive, is currently receiving regular HIV care including how HIV care/treatment has affected a participants life, where they go for treatment, why they decided to go to that particular site, whether access to treatment has changed over time.  **NOTE: THIS CODE SHOULD ALWAYS BE DOUBLE-CODED WITH "HIV services"* | **I: Are you currently receiving regular HIV care?**  P: Yes, but I only missed an appointment sometime in February when I had a sick child in the hospital fortunately, I had extra drugs to use.  **I: How has receiving HIV care/treatment affected your life?**  P: I have experienced a big change ever since I started on care. I have been conducting my chores as usual. Something about these drugs to note of is that my menstrual flow stopped the moment I was initiated into care. I am therefore just eating and sleeping (*laughter*).  **I: Where do you go for medical care?**  P: Here at Ongo Health Centre.  **I: Why did you decide to seek care at that site?**  P: Because it is closer to home and I do not have to struggle to reach the hospital during my appointments.  **I: Have you ever had to change where you access HIV care at any point since you tested positive?**  P: Yes I started care at Homabay Hospital (about 50km from Ongo) which proved far and I therefore had to change. |
| 28 | HIV care barriers | Discussion with an HIV-positive participant about missing an HIV care appointment, dropping out of care for some time, barriers to care including negative experiences with providers, issues of stigma and discloser, problems with money or transportation, depression, etc. Also, what would make it easier to make appointments.  **NOTE: THIS CODE SHOULD ALWAYS BE DOUBLE-CODED WITH "HIV services"* | **“I: Have you ever missed HIV care appointments, or dropped out of care for a time?**  P: Yes.  **I: What were the circumstances that led this to happen?**  P: It happened that one of my children had TB and whenever he was being attended to, I could hear some FACES staff saying that *“those are HIV positive clients”,* I felt hurt and discriminated upon and I therefore dropped from care. One day the facility in charge himself talked to me and urged me to continue and I did so. What impressed me thereafter is when I heard that even some of the FACES staff who were providing HIV services at the PSC were equally HIV positive. I argued that after all, we are in the same boat.  **I: For about how long was your care interrupted?**  P: For about a month.  **I: What are the main barriers you face for not being able to adhere to drugs?**  P: I used to feel really fatigued when I started using the drugs but ever since the drugs were changed, I have never had any problem.  **I: Have you ever missed HIV care appointments, or dropped out of care since you enrolled again?**  P: No, I can only arrive late for the appointment but I have never missed.” |
| 29 | Partnerships | Participant describes sexual partnerships, marriage relationship, any current sexual behavior history. Also, whether the participant has ever considered his/her HIV risk in relation to that partnership or tested as a couple. | “P: No, ever since I enrolled for HIV care, I have never engaged myself in any other sexual affair.”  “**Can you tell me about the partner you consider to be your primary relationship partner?**  P: Yes there is… she is my wife. She has taken care of my life all this time. I have never developed an eye for any woman. In fact at my age, I cannot even go for another sexual partner apart from her.  **I: How did you meet her?**  P: (*laughter*)…I was schooling while staying with my sister, she too was schooling at a neighboring school so we started befriending each other and we later got married immediately after school in 1980. People complained about her age (*very young*) at the time but we have lived peacefully since then.  **I: Do you and your wife ever talk about HIV and the risks of HIV?**  P: It is good to speak the truth…I usually talk to the whole family about HIV. It proved difficult at first but I had to play my parental role. I have been telling them that the current social life is complicated and they have to think twice if they really want to see the growth of their generation. “*If you would like to live with drugs, it is upon you to think about your life”.* My younger son has been my worry and just waiting to test him again.  **I: Do you feel that you are at risk for HIV because of your wife?**  P: I cannot say “*it is impossible*” God knows her better than I do. I have a feeling that she is elderly and she is past that.  **I: Please tell me about anything you are doing in your relationship to try to minimize this risk, or if you don’t feel able to do anything, please tell me about that.**  P: We do talk about HIV and its transmission in our family. We are also happy and ready to be tested at any time.  **I: How has the availability of drugs called antiretrovirals to treat HIV affected this?**  P: Not in any way.  **I: Is there anything else you would like to do in your relationship to minimize your risk, yet you feel you are unable to do?**  P: None, but if in case we get infected, we have to do something…enrolling for care.” |
| 30 | SEARCH concerns | Any discussion of thoughts or concerns about upcoming/ongoing SEARCH activities | I: About Kameke, it is the anxiety people have following the earlier preparatory meetings that were held some time back about the campaigns. People have been asking "but when will the campaigns come".  I: Mmmmmh! “When in deed".  P: And when you are to tell them that "they will come soon", do you know what they will tell you? They will say "we wish it were right now". They so much want the program to get started.  I: Sure.  P: I also think the program of your organization has delayed.  I: You mean people want this program that much?  P: Very. I tell you they have waited for long.  I: You are right about the delay. It is because the IDRC staffs are in another community at the moment carrying out the campaigns and when time for Kameke reaches as per the program line up, I am sure they will be here. By June they will have finished with the campaigns in Kameke and will move to Kadama. So just hold a little patience as you continue encouraging people to be patient too and come in full gear when that time reaches. Isn't it?  P: Yes.  I: Why I say so is because this program is real and so people should not lose hope. (Pause)So that was about the campaigns yet to come. |
