# Supplementary material for: Exploring HIV risk perception mechanisms among youth in a test-and-treat trial in Kenya and Uganda
Source: PLOS Glob Public Health. 2024 May 2;4(5):e0002922. doi: 10.1371/journal.pgph.0002922 (PMC11065277; doi:10.1371/journal.pgph.0002922)
Supplement: S2 File — (DOCX) [file pgph.0002922.s002.docx]

**Informing Pre-Exposure Prophylaxis (PrEP) interventions in SEARCH:**

**FOCUS GROUP DISCUSSION (FGD) GUIDES**

Introduction: To inform the design and implementation of targeted PrEP for sub-populations at high risk of HIV in SEARCH communities, the SEARCH qualitative team will conduct focus group discussions (FGDs) with members of sub-populations determined to be at high HIV risk in SEARCH communities. The aims of this research are to assess attitudes, beliefs, social group norms related to PrEP as well as to gain target population insights into potential implementation strategies.

The teams will conduct gender-specific FGD groups comprised of 8-12 individuals, for each sub-population to be targeted with PrEP activities in each community. The FGD Guides for each of the groups are thematically similar, but tailored to the specific characteristics of each group. This document includes FGD Guides for the following groups:

- FGD Guide A: Adolescent females (ages 15 to 24)
- FGD Guide B: Adolescent males (ages 15 to 24)
- FGD Guide C: Female HIV-Positive Members of HIV-Discordant Couples
- FGD Guide D: Male HIV-Positive Members of HIV-Discordant Couples
- FGD Guide E: Female HIV-Negative Members of HIV-Discordant Couples
- FGD Guide F: Male HIV-Negative Members of HIV-Discordant Couples

The guides include specific probes and follow-up questions, but interviewers will insert additional probes and follow up questions as needed in order to clarify participants’ responses or further explore discussion points that are salient to the research questions.

*Note to interviewers: Instructions for interviewers are in italicized text; the script to guide questioning is in plain text. Do not read FGD guide section headers aloud to participants.*

The Informed Consent process for the PrEP FGDs will be conducted in two stages: the purpose of the research will be communicated to sampled individuals during recruitment. Individual Informed Consent to participate in the FGD can be obtained during the sign-in for the FDG or prior to the FGD. In addition, the FGD Facilitator will recap the elements of the informed consent process, review ground rules for confidentiality among participants, and recap the purpose of the FGD, prior to the start of the discussion. *Please refer to the recruitment checklist document at the end of this guide for more details.*

**FGD Guide A: Adolescent Females (ages 15 to 24)**

My name is ___________________. My colleague who is helping with note-taking today is ___________________. We work for the SEARCH study as researchers.Thank you for taking the time to talk with us today. I would like to ask you some questions about your opinions and experiences as young womenin this community.

We will be discussing what you have heard and what you think about a new HIV prevention method called **PrEP**, which is short for Pre-Exposure Prophylaxis. PrEP is an oral medication (a daily pill) that HIV-negative people can take in order to prevent becoming infected with HIV, if they have unprotected sex with someone who is HIV-positive. **Can I have a show of hands of those of you who have heard of PrEP?** *Note how many have not heard of PrEP and adjust script accordingly.* We will discuss PrEP in more detail shortly.

The information that you provide will be used to inform efforts to improve HIV prevention programs and services for young women like you in [*Uganda*/ *Kenya*] and other countries. Please stop me and let me know if you do not understand any of my questions, so that I can be sure to explain them well. Your complete, honest answers will help us understand your interests, preferences and opinions.

As we discussed in the Informed Consent process, we will be tape recording this discussion. This is so that we can obtain complete and accurate answers, and can transcribe and translate the discussion so that the information that you share with us can be used to inform HIV prevention programs. No names or any other personal identifiers will be attached to either this audio recording or any other documents related to this discussion.

You, as participants, should also respect the confidentiality of what is discussed in this focus group. That means, what is said in this room, stays in this room. Does everyone agree to this ground rule? *Ask for a show of hands and make sure there is consensus re: confidentiality.*

Are there any other ground rules that you suggest that the group should follow? *Probe for other ground rules such as: listening, respect for others’ opinions, etc. Recorder should write Ground Rules down on an easel pad/ large paper on wall for all to see.*

Thank you again for agreeing to participate in this discussion. Do you have any questions? Let’s begin.

1. **HIV risk perceptions**
2. I’d like to start by asking you all to think about how most young women in this community think about their risks of becoming infected with HIV. How do most young women who are HIV-negative, or who don’t know their HIV status, think about their risks of getting infected with HIV?
3. To what extent do young women talk about how they feel about their HIV risk with other people, or do they keep it to themselves?
4. *If participants mention talking about HIV, follow with:* with whom do you feel most comfortable talking about HIV risks? Why do you feel most comfortable talking with them?
5. In your groups of friends, and people you know well, how much concern do people have about their risk of getting infected with HIV?
6. What do you think about that? *Probe for whether or not participants feel this level of risk perception is accurate.*
7. What do you think young women can do to reduce their risks of getting infected with HIV? *Probe for HIV prevention methods, e.g. PrEP, condoms, reducing number of partners, etc.*
8. To what extent do most young women you know do these things [*repeat what participants said/ use the words they use to describe HIV prevention methods*]
9. What can interfere with their ability to do these things? [*use participants’ words*]
10. What about among HIV-positive people you know: what do most people you know do, to reduce risks of spreading the virus to others?
11. Let me now talk with you about anti-retroviral therapy, or ART. Can you tell me, what do you know about ART medications?
12. Earlier in our discussion, we talked about how HIV negative people feel about their risk of getting infected with HIV. Has this changed since ARTs were introduced?
13. If so, how has this changed?
14. Earlier in the discussion, we talked about how HIV positive people feel about their risk of transmitting HIV. Has this changed since ARTs were introduced?
15. If so, how has this changed?
16. **Knowledge and beliefs related to PrEP**
17. Now I want to talk with you about something called PrEP, which as I mentioned, is short for “Pre-Exposure Prophylaxis”. Can you tell me please, what, have you heard about PrEP? [*follow with short educational session; answer any questions participants have*]
18. Where did you learn this information?
19. What do you think most young women in this community know about PrEP?
20. What do they NOT know about PrEP?
21. Have you heard any rumors about PrEP? If so, what have you heard?
22. What do you think might lead young women to want to take PrEP?
23. What other motivations might they have? *Probe for ALL potential motivations*.
24. Who do you think PrEP would be especially helpful for, in this community—what kinds of people?
25. Are there any other groups of people who would benefit from taking PrEP, in this community?
26. What concerns do you have about PrEP? What concerns do you think other young women in this community might have about PrEP?
27. What do you think young women need to know about PrEP, in order to feel comfortable taking PrEP?
28. What other things might young women need in place in their life, in order to feel comfortable taking PrEP?
29. **Concerns and recommendations related to PrEP interventions in this group**
30. Now I want to invite you to share your thoughts and opinions with us about how PrEP can best be offered to young women like you. Let me start with a general question: what do you think the SEARCH study team needs to know, about helping young women in this community to access PrEP?
31. How could information about PrEP best be shared with young women in this community?
32. Where do you think most young women would want to learn about PrEP?
33. Are there ways that information about PrEP should NOT be shared or communicated?
34. How can the groups of people in this community who would most benefit from PrEP best get access to it, without becoming stigmatized?
35. What are the best ways to encourage people who are at risk of HIV to access PrEP?
36. Are there certain groups of people in this community who need a different approach?
37. Are there any other suggestions you have for the SEARCH team about how best to introduce PrEP to people in this community?
38. Now I’d like you to share any additional information you’d like to share with me, about your thoughts or opinions about PrEP or HIV prevention in this community. This could be additional thoughts you have about things we’ve been discussing or something new that we haven’t yet discussed. *Take time for additional comments before closing.*

**Thank you for participating in this study! We appreciate your time and contributions.**

**FGD Guide B: Adolescent Males (ages 15 to 24)**

My name is ___________________. My colleague who is helping with note-taking today is ___________________. We work for the SEARCH study as researchers. Thank you for taking the time to talk with us today. I would like to ask you some questions about your opinions and experiences as young men in this community.

We will be discussing what you have heard and what you think about a new HIV prevention method called **PrEP**, which is short for Pre-Exposure Prophylaxis. PrEP is an oral medication (a daily pill) that HIV-negative people can take in order to prevent becoming infected with HIV, if they have unprotected sex with someone who is HIV-positive. **Can I have a show of hands of those of you who have heard of PrEP?** *Note how many have not heard of PrEP and adjust script accordingly.* We will discuss PrEP in more detail shortly.

The information that you provide will be used to inform efforts to improve HIV prevention programs and services for young men like you in [*Uganda*/ *Kenya*] and other countries. Please stop me and let me know if you do not understand any of my questions, so that I can be sure to explain them well. Your complete, honest answers will help us understand your interests, preferences and opinions.

As we discussed in the Informed Consent process, we will be tape recording this discussion. This is so that we can obtain complete and accurate answers, and can transcribe and translate the discussion so that the information that you share with us can be used to inform HIV prevention programs. No names or any other personal identifiers will be attached to either this audio recording or any other documents related to this discussion.

You, as participants, should also respect the confidentiality of what is discussed in this focus group. That means, what is said in this room, stays in this room. Does everyone agree to this ground rule? *Ask for a show of hands and make sure there is consensus re: confidentiality.*

Are there any other ground rules that you suggest that the group should follow? *Probe for other ground rules such as: listening, respect for others’ opinions, etc. Recorder should write Ground Rules down on an easel pad/ large paper on wall for all to see.*

Thank you again for agreeing to participate in this discussion. Do you have any questions? Let’s begin.

1. **HIV risk perceptions**
2. I’d like to start by asking you all to think about how most young men in this community think about their risks of becoming infected with HIV. How do most young men who are HIV-negative, or who don’t know their HIV status, think about their risks of getting infected with HIV?
3. To what extent do young men talk about how they feel about their HIV risk with other people, or do they keep it to themselves?
4. *If participants mention talking about HIV, follow with:* with whom do you feel most comfortable talking about HIV risks? Why do you feel most comfortable talking with them?
5. In your groups of friends, and people you know well, how much concern do people have about their risk of getting infected with HIV?
6. What do you think about that? *Probe for whether or not participants feel this level of risk perception is accurate.*
7. What do you think young men can do to reduce their risks of getting infected with HIV? *Probe for HIV prevention methods, e.g. PrEP, condoms, reducing number of partners, etc.*
8. To what extent do most young men you know do these things? [*repeat what participants said/ use the words they use to describe HIV prevention methods*]
9. What can interfere with their ability to do these things? [*use participants’ words*]
10. What about among HIV-positive people you know: what do most people you know do, to reduce risks of spreading the virus to others?
11. Let me now talk with you about anti-retroviral therapy, or ART. Can you tell me, what do you know about ART medications?
12. Earlier in our discussion, we talked about how HIV negative people feel about their risk of getting infected with HIV. Has this changed since ARTs were introduced?
13. If so, how has this changed?
14. Earlier in the discussion, we talked about how HIV positive people feel about their risk of transmitting HIV. Has this changed since ARTs were introduced?
15. If so, how has this changed?
16. **Knowledge and beliefs related to PrEP**
17. Now I want to talk with you about something called PrEP, which as I mentioned, is short for “Pre-Exposure Prophylaxis”. Can you tell me please, what have you heard about PrEP? [*follow with short educational session; answer any questions participants have*]
18. Where did you learn this information?
19. What do you think most young men in this community know about PrEP?
20. What do they NOT know about PrEP?
21. Have you heard any rumors about PrEP? If so, what have you heard?
22. What do you think might lead young men to want to take PrEP?
23. What other motivations might they have? *Probe for ALL potential motivations*.
24. Who do you think PrEP would be especially helpful for, in this community—what kinds of people?
25. Are there any other groups of people who would benefit from taking PrEP, in this community?
26. What concerns do you have about PrEP? What concerns do you think other young men in this community might have about PrEP?
27. What do you think young men need to know about PrEP, in order to feel comfortable taking PrEP?
28. What other things might young men need in place in their life, in order to feel comfortable taking PrEP?
29. **Concerns and recommendations related to PrEP interventions in this group**
30. Now I want to invite you to share your thoughts and opinions with us about how PrEP can best be offered to young men like you. Let me start with a general question: what do you think the SEARCH study team needs to know, about helping young men in this community to access PrEP?
31. How could information about PrEP best be shared with young men in this community?
32. Where do you think most young men would want to learn about PrEP?
33. Are there ways that information about PrEP should NOT be shared or communicated?
34. How can the groups of people in this community who would most benefit from PrEP best get access to it, without becoming stigmatized?
35. What are the best ways to encourage people who are at risk of HIV to access PrEP?
36. Are there certain groups of people in this community who need a different approach?
37. Are there any other suggestions you have for the SEARCH team about how best to introduce PrEP to people in this community?
38. Now I’d like you to share any additional information you’d like to share with me, about your thoughts or opinions about PrEP or HIV prevention in this community. This could be additional thoughts you have about things we’ve been discussing or something new that we haven’t yet discussed. *Take time for additional comments before closing.*

**Thank you for participating in this study! We appreciate your time and contributions.**

**FGD Guide C: Female HIV-Positive Members of HIV-Discordant Couples**

My name is ___________________. My colleague who is helping with note-taking today is ___________________. We work for the SEARCH study as researchers.Thank you for taking the time to talk with us today. I would like to ask you some questions about your opinions and experiences as HIV-positive women in this community who are in HIV-discordant couples.

We will be discussing what you have heard and what you think about a new HIV prevention method called **PrEP**, which is short for Pre-Exposure Prophylaxis. PrEP is an oral medication (a daily pill) that HIV-negative people can take in order to prevent becoming infected with HIV, if they have unprotected sex with someone who is HIV-positive. **Can I have a show of hands of those of you who have heard of PrEP?** *Note how many have not heard of PrEP and adjust script accordingly.* We will discuss PrEP in more detail shortly.

The information that you provide will be used to inform efforts to improve HIV prevention programs and services in [*Uganda*/ *Kenya*] and other countries. Please stop me and let me know if you do not understand any of my questions, so that I can be sure to explain them well. Your complete, honest answers will help us understand your interests, preferences and opinions.

As we discussed in the Informed Consent process, we will be tape recording this discussion. This is so that we can obtain complete and accurate answers, and can transcribe and translate the discussion so that the information that you share with us can be used to inform HIV prevention programs. No names or any other personal identifiers will be attached to either this audio recording or any other documents related to this discussion.

You, as participants, should also respect the confidentiality of what is discussed in this focus group. That means, what is said in this room, stays in this room. Does everyone agree to this ground rule? *Ask for a show of hands and make sure there is consensus re: confidentiality.*

Are there any other ground rules that you suggest that the group should follow? *Probe for other ground rules such as: listening, respect for others’ opinions, etc. Recorder should write Ground Rules down on an easel pad/ large paper on wall for all to see.*

Thank you again for agreeing to participate in this discussion. Do you have any questions? Let’s begin.

1. **HIV risk perceptions and disclosure experiences**
2. I’d like to start by asking you all to think about how most women in this community think about their risks of becoming infected with HIV. How do most women who are HIV-negative, or who don’t know their HIV status, think about their risks of getting infected with HIV?
3. To what extent do women talk about how they feel about their HIV risk with other people, or do they keep it to themselves? Who do they confide in, most often? Why?
4. How about the men in this community who are HIV-negative, or who don’t know their status—what do they think about their risks of getting infected with HIV?
5. To what extent do you think they talk about their risks with others? Who do you think most men tend to confide in, in your opinion? Why?
6. Now I want to ask you about women like you who are HIV-positive. To what extent are you worried about your risk of transmitting HIV to your partner?
7. What about other HIV-positive women in this community—to what extent do you feel most HIV-positive women are worried about transmitting HIV to their partner?
8. Do you feel it’s the same, or different, for men who are HIV-positive in this community? *Pause for responses.* Please tell me more about that—what do others think?
9. Now I want to ask you some questions about your experiences as members of HIV-discordant couples, and about the challenges women face related to disclosing their HIV status.

Sometimes women in marital relationships, or in non-marital sexual relationships, struggle with the decision whether or not to tell a partner that they are HIV-positive. Tell me, what are some of the things that HIV-positive women worry about, related to disclosing their HIV-positive status to their partner?

1. Can you tell us about your experiences talking with your spouse/partner(s) about your HIV status?
   - And how did you feel about that decision?
   - How about others—have any of you had similar experiences?
   - Have any of you had different experiences? Please tell us about that.
2. What makes it difficult for women to talk with their partner/spouse about their HIV status?
   - What would help make it easier for women to talk with their partner about their HIV status?
3. What do you think HIV-positive people in HIV-discordant couples can do to reduce their risks of transmitting HIV to their partners? *Probe for HIV prevention methods, e.g. PrEP, condoms, reducing number of partners, adhering to ART, etc.*
4. To what extent do you think most people in discordant couples do these things? [*repeat what participants said/ use the words they use to describe HIV prevention methods*]
5. What can interfere with their ability to do these things? [*use participants’ words*]
6. Let me now talk with you about anti-retroviral therapy, or ART. Can you tell me, what do you know about ART medications?
7. What are the main motivations that HIV-positive women have for taking ART?
8. What worries or concerns do women have, about starting ART medications?
9. Tell me, please, for those of you who are taking ART, How has taking ARTs affected your life?
10. What have you been told about how ART can help reduce your risk of transmitting HIV to others?
11. Please tell me about any challenges women may have with adhering to HIV medications? *Probe for ALL reasons.*
12. What would make it easier for women who are facing these challenges, to adhere to taking HIV medications?
13. Earlier in our discussion, we talked about how HIV negative people feel about their risk of getting infected with HIV. Has this changed since ARTs were introduced?
14. If so, how has this changed?
15. Earlier in the discussion, we talked about how HIV positive people feel about their risk of transmitting HIV. Has this changed since ARTs were introduced?
16. If so, how has this changed?
17. **Knowledge and beliefs related to PrEP**
18. Now I want to talk with you about something called PrEP, which as I mentioned, is short for “Pre-Exposure Prophylaxis”. Can you tell me please, what have you heard about PrEP? [*follow with short educational session; answer any questions participants have*]
19. Where did you learn this information?
20. What do you think most women in this community know about PrEP?
21. What do they NOT know about PrEP?
22. Have you heard any rumors about PrEP? If so, what have you heard?
23. What do you think might lead people to want to take PrEP?
24. What other motivations might they have? *Probe for ALL potential motivations*.
25. Who do you think PrEP would be especially helpful for, in this community—what kinds of people?
26. Are there any other groups of people who would benefit from taking PrEP, in this community?
27. What do you think HIV-negative members of discordant couples need to know about PrEP, in order to feel comfortable taking PrEP?
28. What other things might HIV-negative members of discordant couples need in place in their life, in order to feel comfortable taking PrEP?
29. **Concerns and recommendations related to PrEP interventions in this group**
30. Now I want to invite you to share your thoughts and opinions with us about how PrEP can best be offered to HIV-discordant couples. Let me start with a general question: what do you think the SEARCH study team needs to know, about helping HIV-positive women and their partners in this community to access PrEP?
31. How could information about PrEP best be shared with people in discordant couples in this community?
32. Where do you think most people in discordant couples would want to learn about PrEP?
33. Are there ways that information about PrEP should NOT be shared or communicated?
34. How can the groups of people in this community who would most benefit from PrEP best get access to it, without becoming stigmatized?
35. How can HIV-positive women in discordant couples best be supported, with accessing ART and PrEP for their partners?
36. What are the best ways to encourage people who are at risk of HIV to access PrEP?
37. Are there certain groups of people in this community who need a different approach?
38. Are there any other suggestions you have for the SEARCH team about how best to introduce PrEP to people in HIV-discordant couples, or at risk of HIV, in this community?
39. Now I’d like you to share any additional information you’d like to share with me, about your thoughts or opinions about PrEP or HIV prevention in this community. This could be additional thoughts you have about things we’ve been discussing or something new that we haven’t yet discussed. *Take time for additional comments before closing.*

**Thank you for participating in this study! We appreciate your time and contributions.**

**FGD Guide D: Male HIV-Positive Members of HIV-Discordant Couples**

My name is ___________________. My colleague who is helping with note-taking today is ___________________. We work for the SEARCH study as researchers.Thank you for taking the time to talk with us today. I would like to ask you some questions about your opinions and experiences as HIV-positive men in this community who are in HIV-discordant couples.

We will be discussing what you have heard and what you think about a new HIV prevention method called **PrEP**, which is short for Pre-Exposure Prophylaxis. PrEP is an oral medication (a daily pill) that HIV-negative people can take in order to prevent becoming infected with HIV, if they have unprotected sex with someone who is HIV-positive. **Can I have a show of hands of those of you who have heard of PrEP?** *Note how many have not heard of PrEP and adjust script accordingly.* We will discuss PrEP in more detail shortly.

The information that you provide will be used to inform efforts to improve HIV prevention programs and services in [*Uganda*/ *Kenya*] and other countries. Please stop me and let me know if you do not understand any of my questions, so that I can be sure to explain them well. Your complete, honest answers will help us understand your interests, preferences and opinions.

As we discussed in the Informed Consent process, we will be tape recording this discussion. This is so that we can obtain complete and accurate answers, and can transcribe and translate the discussion so that the information that you share with us can be used to inform HIV prevention programs. No names or any other personal identifiers will be attached to either this audio recording or any other documents related to this discussion.

You, as participants, should also respect the confidentiality of what is discussed in this focus group. That means, what is said in this room, stays in this room. Does everyone agree to this ground rule? *Ask for a show of hands and make sure there is consensus re: confidentiality.*

Are there any other ground rules that you suggest that the group should follow? *Probe for other ground rules such as: listening, respect for others’ opinions, etc. Recorder should write Ground Rules down on an easel pad/ large paper on wall for all to see.*

Thank you again for agreeing to participate in this discussion. Do you have any questions? Let’s begin.

1. **HIV risk perceptions and disclosure experiences**
2. I’d like to start by asking you all to think about how most men in this community think about their risks of becoming infected with HIV. How do most men who are HIV-negative, or who don’t know their HIV status, think about their risks of getting infected with HIV?
3. To what extent do men talk about how they feel about their HIV risk with other people, or do they keep it to themselves? Who do they confide in, most often? Why?
4. How about the women in this community who are HIV-negative, or who don’t know their status—what do they think about their risks of getting infected with HIV?
5. To what extent do you think they talk about their risks with others? Who do you think most women tend to confide in, in your opinion? Why?
6. Now I want to ask you about men like you who are HIV-positive. To what extent are you worried about your risk of transmitting HIV to your partner?
7. What about other HIV-positive men in this community—to what extent do you feel most HIV-positive men are worried about transmitting HIV to their partner?
8. Do you feel it’s the same, or different, for women who are HIV-positive in this community? *Pause for responses.* Please tell me more about that—what do others think?
9. Now I want to ask you some questions about your experiences as members of HIV-discordant couples, and about the challenges men face related to disclosing their HIV status.

Sometimes men in marital relationships, or in non-marital sexual relationships, struggle with the decision whether or not to tell a partner that they are HIV-positive. Tell me, what are some of the things that HIV-positive men worry about, related to disclosing their HIV-positive status to their partner?

1. Can you tell us about your experiences talking with your spouse/partner(s) about your HIV status?
   - And how did you feel about that decision?
   - How about others—have any of you had similar experiences?
   - Have any of you had different experiences? Please tell us about that.
2. What makes it difficult for men to talk with their partner/spouse about their HIV status?
   - What would help make it easier for men to talk with their partner about their HIV status?
3. What do you think HIV-positive people in HIV-discordantcouples can do to reduce their risks of transmitting HIV to their partners? *Probe for HIV prevention methods, e.g. PrEP, condoms, reducing number of partners, adhering to ART, etc.*
4. To what extent do you think most people in discordant couples do these things? [*repeat what participants said/ use the words they use to describe HIV prevention methods*]
5. What can interfere with their ability to do these things? [*use participants’ words*]
6. Let me now talk with you about anti-retroviral therapy, or ART. Can you tell me, what do you know about ART medications?
7. What are the main motivations that HIV-positive men have for taking ART?
8. What worries or concerns do men have, about starting ART medications?
9. Tell me, please, for those of you who are taking ART, How has taking ARTs affected your life?
10. What have you been told about how ART can help reduce your risk of transmitting HIV to others?
11. Please tell me about any challenges men may have with adhering to HIV medications? *Probe for ALL reasons.*
12. What would make it easier for men who are facing these challenges, to adhere to taking HIV medications?
13. Earlier in our discussion, we talked about how HIV negative people feel about their risk of getting infected with HIV. Has this changed since ARTs were introduced?
14. If so, how has this changed?
15. Earlier in the discussion, we talked about how HIV positive people feel about their risk of transmitting HIV. Has this changed since ARTs were introduced?
16. If so, how has this changed?
17. **Knowledge and beliefs related to PrEP**
18. Now I want to talk with you about something called PrEP, which as I mentioned, is short for “Pre-Exposure Prophylaxis”. Can you tell me please, what have you heard about PrEP? [*follow with short educational session; answer any questions participants have*]
19. Where did you learn this information?
20. What do you think most men in this community know about PrEP?
21. What do they NOT know about PrEP?
22. Have you heard any rumors about PrEP? If so, what have you heard?
23. What do you think might lead people to want to take PrEP?
24. What other motivations might they have? *Probe for ALL potential motivations*.
25. Who do you think PrEP would be especially helpful for, in this community—what kinds of people?
26. Are there any other groups of people who would benefit from taking PrEP, in this community?
27. What do you think HIV-negative members of discordant couples need to know about PrEP, in order to feel comfortable taking PrEP?
28. What other things might HIV-negative members of discordant couplesneed in place in their life, in order to feel comfortable taking PrEP?
29. **Concerns and recommendations related to PrEP interventions in this group**
30. Now I want to invite you to share your thoughts and opinions with us about how PrEP can best be offered to HIV-discordant couples. Let me start with a general question: what do you think the SEARCH study team needs to know, about helping HIV-positive men and their partners in this community to access PrEP?
31. How could information about PrEP best be shared with people in discordant couples in this community?
32. Where do you think most people in discordant couples would want to learn about PrEP?
33. Are there ways that information about PrEP should NOT be shared or communicated?
34. How can the groups of people in this community who would most benefit from PrEP best get access to it, without becoming stigmatized?
35. How can HIV-positive men in discordant couples best be supported, with accessing ART and PrEP for their partners?
36. What are the best ways to encourage people who are at risk of HIV to access PrEP?
37. Are there certain groups of people in this community who need a different approach?
38. Are there any other suggestions you have for the SEARCH team about how best to introduce PrEP to people in HIV-discordant couples, or at risk of HIV, in this community?
39. Now I’d like you to share any additional information you’d like to share with me, about your thoughts or opinions about PrEP or HIV prevention in this community. This could be additional thoughts you have about things we’ve been discussing or something new that we haven’t yet discussed. *Take time for additional comments before closing.*

**Thank you for participating in this study! We appreciate your time and contributions.**

**FGD Guide E: Female HIV-Negative Members of HIV-Discordant Couples**

My name is ___________________. My colleague who is helping with note-taking today is ___________________. We work for the SEARCH study as researchers.Thank you for taking the time to talk with us today. I would like to ask you some questions about your opinions and experiences as women in this community who are HIV-negative members of HIV-discordant couples.

We will be discussing what you have heard and what you think about a new HIV prevention method called **PrEP**, which is short for Pre-Exposure Prophylaxis. PrEP is an oral medication (a daily pill) that HIV-negative people can take in order to prevent becoming infected with HIV, if they have unprotected sex with someone who is HIV-positive. **Can I have a show of hands of those of you who have heard of PrEP?** *Note how many have not heard of PrEP and adjust script accordingly.* We will discuss PrEP in more detail shortly.

The information that you provide will be used to inform efforts to improve HIV prevention programs and services for women like you in [*Uganda*/ *Kenya*] and other countries. Please stop me and let me know if you do not understand any of my questions, so that I can be sure to explain them well. Your complete, honest answers will help us understand your interests, preferences and opinions.

As we discussed in the Informed Consent process, we will be tape recording this discussion. This is so that we can obtain complete and accurate answers, and can transcribe and translate the discussion so that the information that you share with us can be used to inform HIV prevention programs. No names or any other personal identifiers will be attached to either this audio recording or any other documents related to this discussion.

You, as participants, should also respect the confidentiality of what is discussed in this focus group. That means, what is said in this room, stays in this room. Does everyone agree to this ground rule? *Ask for a show of hands and make sure there is consensus re: confidentiality.*

Are there any other ground rules that you suggest that the group should follow? *Probe for other ground rules such as: listening, respect for others’ opinions, etc. Recorder should write Ground Rules down on an easel pad/ large paper on wall for all to see.*

Thank you again for agreeing to participate in this discussion. Do you have any questions? Let’s begin.

1. **HIV risk perceptions**
2. I’d like to start by asking you all to think about how most women in this community think about their risks of becoming infected with HIV. How do most women who are HIV-negative, or who don’t know their HIV status, think about their risks of getting infected with HIV?
3. To what extent do women talk about how they feel about their HIV risk with other people, or do they keep it to themselves?
4. *If participants mention talking about HIV, follow with:*With whom do you feel most comfortable talking about HIV risks? Why do you feel most comfortable talking with them?
5. In your groups of friends, and people you know well, how much concern do people have about their risk of getting infected with HIV?
6. What do you think about that? *Probe for whether or not participants feel this level of risk perception is accurate.*
7. What do you think women can do to reduce their risks of getting infected with HIV? *Probe for HIV prevention methods, e.g. PrEP, condoms, reducing number of partners, etc.*
8. To what extent do most women you know do these things [*repeat what participants said/ use the words they use to describe HIV prevention methods*]
9. What can interfere with their ability to do these things? [*use participants’ words*]
10. What about among HIV-positive people you know: what do most people you know do, to reduce risks of spreading the virus to others?
11. Let me now talk with you about anti-retroviral therapy, or ART. Can you tell me, what do you know about ART medications?
12. Tell me please, how do you think access to ART is affecting how people in this community feel about their risk of getting infected with HIV?
13. Specifically, how it is affecting women, if at all?
14. How about people who are HIV-positive in this community: how do you think taking ART is affecting how people think about their risk of transmitting HIV to other people?
15. Specifically, how it is affecting women, if at all?
16. Now I want to ask you about your experiences as HIV-negative women who have partners who are HIV-positive. Please tell me, how did you come to learn about your HIV-discordant couples status?
17. Would you have preferred to learn about it in another way? Please tell me more about that.
18. Being an HIV-negative person in a relationship with an HIV-positive person can be challenging experience. Please tell me, what are the main challenges, for you?
19. What are the things that can motivate HIV-negative people to stay committed to their HIV-positive partners despite their risks of HIV infection?
20. What are the things that HIV-positive people can do, to help protect and support their HIV-negative partners?
21. **Knowledge and beliefs related to PrEP**
22. Now I want to talk with you about something called PrEP, which as I mentioned, is short for “Pre-Exposure Prophylaxis”. Can you tell me please, what have you heard about PrEP? [*follow with short educational session; answer any questions participants have*]
23. Where did you learn this information?
24. What do you think most people in HIV-discordant relationships in this community know about PrEP?
25. What do they NOT know about PrEP?
26. Have you heard any rumors about PrEP? If so, what have you heard?
27. What do you think might lead women to want to take PrEP?
28. What other motivations might they have? *Probe for ALL potential motivations*.
29. Who do you think PrEP would be especially helpful for, in this community—what kinds of people?
30. Are there any other groups of people who would benefit from taking PrEP, in this community?
31. What concerns do you have about PrEP? What concerns do you think other women in this community might have about PrEP?
32. What do you think women at risk of HIV infection need to know about PrEP, in order to feel comfortable taking PrEP?
33. What other things might women need in place in their life, in order to feel comfortable taking PrEP?
34. Have any of you had experiences with taking PrEP? *If yes*: Please share with us any experiences you have had so far.
35. What were your main motivations for starting PrEP?
36. How has it affected your feelings about your risk of acquiring HIV infection?
37. How has it affected your relationship with your partner? *Probe for whether others have had similar, or different experiences.*
38. **Concerns and recommendations related to PrEP interventions in this group**
39. Now I want to invite you to share your thoughts and opinions with us about how PrEP can best be offered to women like you who are in HIV-discordant couples. Let me start with a general question: what do you think the SEARCH study team needs to know, about helping women in this community to access PrEP?
40. How could information about PrEP best be shared with women and their partners in this community?
41. Where do you think most women in HIV-discordant couples would want to learn about PrEP?
42. Are there ways that information about PrEP should NOT be shared or communicated?
43. How can the groups of people in this community who would most benefit from PrEP best get access to it, without becoming stigmatized?
44. What are the best ways to encourage people who are at risk of HIV to access PrEP?
45. Are there certain groups of people in this community who need a different approach?
46. Are there any other suggestions you have for the SEARCH team about how best to introduce PrEP to people in HIV-discordant couples in this community?
47. Now I’d like you to share any additional information you’d like to share with me, about your thoughts or opinions about PrEP or HIV prevention in this community. This could be additional thoughts you have about things we’ve been discussing or something new that we haven’t yet discussed. *Take time for additional comments before closing.*

**Thank you for participating in this study! We appreciate your time and contributions.**

**FGD Guide F: Male HIV-Negative Members of HIV-Discordant Couples**

My name is ___________________. My colleague who is helping with note-taking today is ___________________. We work for the SEARCH study as researchers.Thank you for taking the time to talk with us today. I would like to ask you some questions about your opinions and experiences as men in this community who are HIV-negative members of HIV-discordant couples.

We will be discussing what you have heard and what you think about a new HIV prevention method called **PrEP**, which is short for Pre-Exposure Prophylaxis. PrEP is an oral medication (a daily pill) that HIV-negative people can take in order to prevent becoming infected with HIV, if they have unprotected sex with someone who is HIV-positive. **Can I have a show of hands of those of you who have heard of PrEP?** *Note how many have not heard of PrEP and adjust script accordingly.* We will discuss PrEP in more detail shortly.

The information that you provide will be used to inform efforts to improve HIV prevention programs and services for men like you in [*Uganda*/ *Kenya*] and other countries. Please stop me and let me know if you do not understand any of my questions, so that I can be sure to explain them well. Your complete, honest answers will help us understand your interests, preferences and opinions.

As we discussed in the Informed Consent process, we will be tape recording this discussion. This is so that we can obtain complete and accurate answers, and can transcribe and translate the discussion so that the information that you share with us can be used to inform HIV prevention programs. No names or any other personal identifiers will be attached to either this audio recording or any other documents related to this discussion.

You, as participants, should also respect the confidentiality of what is discussed in this focus group. That means, what is said in this room, stays in this room. Does everyone agree to this ground rule? *Ask for a show of hands and make sure there is consensus re: confidentiality.*

Are there any other ground rules that you suggest that the group should follow? *Probe for other ground rules such as: listening, respect for others’ opinions, etc. Recorder should write Ground Rules down on an easel pad/ large paper on wall for all to see.*

Thank you again for agreeing to participate in this discussion. Do you have any questions? Let’s begin.

1. **HIV risk perceptions**
2. I’d like to start by asking you all to think about how most men in this community think about their risks of becoming infected with HIV. How do most men who are HIV-negative, or who don’t know their HIV status, think about their risks of getting infected with HIV?
3. To what extent do men talk about how they feel about their HIV risk with other people, or do they keep it to themselves?
4. *If participants mention talking about HIV, follow with:*With whom do you feel most comfortable talking about HIV risks? Why do you feel most comfortable talking with them?
5. In your groups of friends, and people you know well, how much concern do people have about their risk of getting infected with HIV?
6. What do you think about that? *Probe for whether or not participants feel this level of risk perception is accurate.*
7. What do you think men can do to reduce their risks of getting infected with HIV? *Probe for HIV prevention methods, e.g. PrEP, condoms, reducing number of partners, etc.*
8. To what extent do most men you know do these things [*repeat what participants said/ use the words they use to describe HIV prevention methods*]
9. What can interfere with their ability to do these things? [*use participants’ words*]
10. What about among HIV-positive people you know: what do most people you know do, to reduce risks of spreading the virus to others?
11. Let me now talk with you about anti-retroviral therapy, or ART. Can you tell me, what do you know about ART medications?
12. Tell me please, how do you think access to ART is affecting how people in this community feel about their risk of getting infected with HIV?
13. Specifically, how it is affecting men, if at all?
14. How about people who are HIV-positive in this community: how do you think taking ART is affecting how people think about their risk of transmitting HIV to other people?
15. Specifically, how it is affecting men, if at all?
16. Now I want to ask you about your experiences as HIV-negative men who have partners who are HIV-positive. Please tell me, how did you come to learn about your HIV-discordant couples status?
17. Would you have preferred to learn about it in another way? Please tell me more about that.
18. Being an HIV-negative person in a relationship with an HIV-positive person is a very challenging experience. Please tell me, what are the main challenges, for you?
19. What are the things that can motivate HIV-negative men to stay committed to their HIV-positive partners despite their risks of HIV infection?
20. What are the things that HIV-positive women can do, to help protect and support their HIV-negative partners?
21. **Knowledge and beliefs related to PrEP**
22. Now I want to talk with you about something called PrEP, which as I mentioned, is short for “Pre-Exposure Prophylaxis”. Can you tell me please, what have you heard about PrEP? [*follow with short educational session; answer any questions participants have*]
23. Where did you learn this information?
24. What do you think most people in HIV-discordant relationships in this community know about PrEP?
25. What do they NOT know about PrEP?
26. Have you heard any rumors about PrEP? If so, what have you heard?
27. What do you think might lead men to want to take PrEP?
28. What other motivations might they have? *Probe for ALL potential motivations*.
29. Who do you think PrEP would be especially helpful for, in this community—what kinds of people?
30. Are there any other groups of people who would benefit from taking PrEP, in this community?
31. What concerns do you have about PrEP? What concerns do you think other men in this community might have about PrEP?
32. What do you think men at risk of HIV infection need to know about PrEP, in order to feel comfortable taking PrEP?
33. What other things might men need in place in their life, in order to feel comfortable taking PrEP?
34. Have any of you had experiences with taking PrEP? *If yes*: Please share with us any experiences you have had so far.
35. What were your main motivations for starting PrEP?
36. How has it affected your feelings about your risk of acquiring HIV infection?
37. How has it affected your relationship with your partner?

*Probe for whether others have had similar, or different experiences.*

1. **Concerns and recommendations related to PrEP interventions in this group**
2. Now I want to invite you to share your thoughts and opinions with us about how PrEP can best be offered to men like you who are in HIV-discordant couples. Let me start with a general question: what do you think the SEARCH study team needs to know, about helping men in this community to access PrEP?
3. How could information about PrEP best be shared with men and their partners in this community?
4. Where do you think most men in HIV-discordant couples would want to learn about PrEP?
5. Are there ways that information about PrEP should NOT be shared or communicated?
6. How can the groups of people in this community who would most benefit from PrEP best get access to it, without becoming stigmatized?
7. What are the best ways to encourage people who are at risk of HIV to access PrEP?
8. Are there certain groups of people in this community who need a different approach?
9. Are there any other suggestions you have for the SEARCH team about how best to introduce PrEP to people in HIV-discordant couples in this community?
10. Now I’d like you to share any additional information you’d like to share with me, about your thoughts or opinions about PrEP or HIV prevention in this community. This could be additional thoughts you have about things we’ve been discussing, or something new that we haven’t yet discussed. *Take time for additional comments before closing.*

Thank you for participating in this study! We appreciate your time and contributions.

**Recruitment Checklist for FGD SEARCH Qualitative Phase II**

The SEARCH FGDs on PrEP include the recruitment of groups requires additional ethical consideration to prevent inadvertent disclosure and ensure the confidentiality and protection of all individuals who participate in the FGDs. Informed consent will occur in two stages:

1. Once eligible have been identified at the CHC, during HBT, or through the clinicians, a member of the qualitative team will contract the individual to invite them to participate in the study.
2. The qualitative team will explain the nature of the discussion, the group, and emphasize that there will be others in the group with similar or discordancy status. This is emphasized for the four FGDs with HIV positive and negative male and female members of discordant relationships.
3. The qualitative team will ask for verbal consent after explaining the he study purpose and before inviting the participant to the FGD.
4. The qualitative team will share the location and details of the FGD only after obtaining verbal consent and determining that the participant has fully understood, and agreed to participate in the FGD.
5. All attendees will go through a group informed consent process after signing into the FGD discussions, and in advance of the session.

**Checklist for FGD recruitment**

| **Items** | **Check when completed** |
| --- | --- |
| 1. Contact the potential participant and explain the study purpose, introduce the FGD, and ask for their time | □ |
| 1. Explain the nature of the FGD and highlight:    1. That other members of discordant relationships will be present    2. That participants may discuss discordancy and their partners    3. That HIV status is disclosed during the group session    4. That others in the group will know the discordancy status of all attendees | □ |
| 1. Gauge the comfort and readiness of the participant to attend the FGD | □ |
| 1. Obtain verbal consent and ask participant to summarize in their own words, the purpose of the group, the attendees, and areas for discussion. | □ |
| 1. Ask the participant if s/he will protect information provided during the FGDs, including the discordancy information/HIV status of other attendees. | □ |
| 1. Provide the participant with information about the session location, time, and date. | □ |
| 1. Conduct the group informed consent process after sign in and with all others. | □ |
