# Supplementary material for: Exploring HIV risk perception mechanisms among youth in a test-and-treat trial in Kenya and Uganda
Source: PLOS Glob Public Health. 2024 May 2;4(5):e0002922. doi: 10.1371/journal.pgph.0002922 (PMC11065277; doi:10.1371/journal.pgph.0002922)
Supplement: Attachment — Submitted filename: PGPH-D-23-02191_review.docx [file pgph.0002922.s004.docx]

**PGPH-D-23-02191 Review**

Well-written paper that adds to the literature around HIV risk perception among young people. The rules guiding the HBM in the scientific study were delicately respected. The quotes are informative and lend valuable insights. A few thoughts to improve the paper.

**Introduction**

**Line 36:** Is there any work from the parent study that was published? It will be good to give the citation here.

**Line 39-40:** Some of these details fit best in the method section. Please clarify if this qualitative study was nested in a larger study given the details provided here.

**Line 64:** After introducing the study, the author goes ahead to describe how other people have used the theory which I think may not be necessary unless the paper aims to improve the tenets of the theory, which is not the case here since the theory is only used to better understand the findings. A brief description of the theory and its application would suffice right before introducing your study. This also helps in being concise and focusing the reader on your work. Other details may be included in the discussion where appropriate.

**Methods**

**Line 130:** Consider describing the high HIV prevalence in the two regions and provide relevant citations.

**Line 132:** Not sure about ‘boda boda’ drivers being a globally universal word.

**Line 134:** Write FGD in full for the first mention.

**Line 135:** Using the word ‘we decided to concentrate on’ may not be very appropriate here. Instead, just mention what the study did e.g. The study used FGDs…and give the rationale for using the FGDs.

**Line 143:** Please clarify if community resident youth are the study participants it is not very clear. This statement ‘the LAM represented’… ‘they were further balanced’…I suppose these were mobilizers, right? So FGD participants were purposively sampled based on?

**Line 150**: What is the rationale for conducting an FGD with up to 17 participants in one sitting? given the threshold is 6-12, and given group dynamics? Why was this FGD not divided into two?

How were the FGDs conducted? i.e. how many moderators and note-takers please clarify.

**Line 152:** Consider the number of FGDs per site and how they were distributed or refer to (table 1).

**Line 154:** Consider giving a brief description of your FGD guide, and what it elicited

**Line 158:** Name table 1.

**Line 161:** 6 researchers? What role did each researcher play in the analysis? The author’s reporting does not adhere to the COREQ guidelines for reporting qualitative work. Check that your methods align with this in your methods.

Were there any intercoder agreements to ensure there was consensus in the coding process, please describe.

Provide citation or web-based link for dedoose software.

**Line 176:** NASCOP, KEMRI, and UNCST say ≥ 12 can consent, please clarify under what circumstances and provide relevant citations.

Also, for clarity, indicate that participants provided written informed consent.

**Results**

Were there any demographic data collected from the FGD participants? e.g. social demographic information?

**Line 183:** Please provide a general summary overview of your findings before expanding the themes.

**Line 161:** You describe 4 tenets in the HBM but only describe 2 in the findings please clarify.

**Discussion**

**Line 571:** Please clarify did you explore the constructs of HBM or did you explore HIV risk mechanisms framed in the context of HBM?

**References**

Some references are rather old e.g. 1991, 2000 Please confirm that there is no recent literature for this work.

Consider providing a web-based link for citations 6,7,8.

Please review your citations, and be sure to use the right referencing tool for uniformity.
