# Supplementary material for: Exploring HIV risk perception mechanisms among youth in a test-and-treat trial in Kenya and Uganda
Source: PLOS Glob Public Health. 2024 May 2;4(5):e0002922. doi: 10.1371/journal.pgph.0002922 (PMC11065277; doi:10.1371/journal.pgph.0002922)
Supplement: Attachment — Submitted filename: Letter of Response to Editors and Reviewers 2024-01-22.docx [file pgph.0002922.s005.docx]

**Manuscript ID:** PGPH-D-23-02191

**Manuscript Title:** Exploring HIV risk perception mechanisms among youth in a test-and-treat trial in Kenya and Uganda

**Dear editors,**

**Thank you for the opportunity to address the helpful comments provided by the reviewers and editors. We have edited the manuscript in response to these comments, and provide both a clean and marked copy of the revised manuscript with this resubmission. We have done our best to undertake a thorough and responsive revision of the manuscript. We hope that the editors and reviewers agree that the revised manuscript is much stronger as a result of your very helpful critiques.**

**Below please find each comment with our response indented and in bold print:**

*Editorial Comments to Author:*

The reviewers and I found the manuscript to be well-written and feel it provides valuable insights into how youth perceive risk in the context of test-and-treat in Kenya and Uganda.

**We thank the editors and reviewers for noting our work.**

I agree with Reviewer 2 (see attached file with detailed comments) that some additional clarification in the methods would be helpful, and that aligning the write-up with COREQ or similar qualitative writing guidelines would improve the manuscript. I would also encourage the authors to include their final data collection and analysis tools (FGD guide, final codebook, etc.) in the supplemental materials.

**We are grateful for both reviewer 2 and the editor’s recommendations. We have attached the FGD guide, codebook, and COREQ guidelines as requested. We have also adjusted the methods section in minor details as noted below as a result.**

I would also encourage the authors to consider and respond to the questions raised by Reviewer 1, which may help clarify and position your findings in the context of the literature.

**We thank the editor for this useful suggestion, and we have taken into consideration each reviewer’s comment as described below.**

*Reviewer 1 Comments to the Author*

Overall this paper is well written and addresses an important issue relating to young people’s HIV risk perception, a critical consideration. I have made a small number of specific comments, and then some more general comments for the authors’ consideration, as well as some recommendations and suggestions for further reading.

**We appreciate Reviewer 1’s observations, which we feel have improved this paper, and their encouraging response to our work.**

- Line 14: this the first mention of PrEP – so the acronym should be explained here at first mention

**We have corrected this oversight and now spell out the acronym in full as ‘pre-exposure prophylaxis’.**

- Line 457: poses?

**We thank the reviewer for the comment, but we did not, however, understand how ‘poses’ fits in line 457 and thus have made no changes.**

- Line 460: check punctuation

**We are grateful for the close eye of Reviewer 1 and have made the correction by removing a full stop and a comma.**

1) It would be interesting to unpack the complexity of risk perception around HIV. Prior research from sub Saharan Africa has shown that young people’s narratives of risk perception can be inconsistent and at times contradictory, suggesting that there are dualistic and contradictory notions of fear of HIV infection on the one hand – linked to the persistent and enduring association between HIV and death, and anticipated judgement, rejection and discrimination, while at the same time and a lack of concern regarding HIV infection linked to the destigmatisation and normalisation of HIV and ARVs. How should programmes and policies balance these 2 notions – while not increasing stigma around HIV, but at the same time ensuring that HIV infection is not something considered trivial.

**We agree with the reviewers and have added the following to the introduction: “Policies which acknowledge this complexity are crucial to achieve the greatest possible reach. We focus specifically on the complexities of risk perception as they relate to youth in the context of the rollout of PrEP and Test and Treat, as unpacking the complexity of youth HIV risk perception in full is beyond the scope of this paper”**

**Additionally, we unpack our findings as they relate to these inconsistencies in the discussion section through an entirely new paragraph: “Finally, some studies have raised the concern that normalizing HIV and ART can have the effect of lowering risk perceptions and consequent preventive behaviours – including testing behaviours [54]. In one study in South Africa, like in ours, the younger cohort feel HIV is a manageable illness today, even though they nonetheless fear getting a positive HIV test result. While that attitude can be interpreted as a contradiction, in the HBM it is explainable by the difference between risk perception and disease severity. While perception of HIV severity is low because it is treatable, risk perception can remain high, especially when the ‘risk’ extends beyond risk of the disease itself to involve the social consequences of living with the disease. Such consequences can include stigma and challenges to one’s social relations (e.g. finding a marriage partner, disclosure, etc). While such social consequences should be considered within the category of ‘severity’, too often a clinical approach focuses only on how a disease affects the body, which is then taken up socially. Broadening a strict disease-focus to include the social scientific recognition that illness is a biopsychosocial phenomenon that extends beyond the mere biomedical diagnosis of a disease means that care must be taken to determine whether lay participants include the social consequences of a diagnosis within perceptions about either severity of a disease or the risks it presents to their (socially-embedded) lives. In our cohort, youth appear to associate social consequences with over-all risk perception (i.e. likelihood of acquiring the infection and what it means socially), rather than severity (i.e. degree to which it affects the body and care of the embodied self).”**

**Finally, we recommend that “Comprehensive sexuality education through trusted and credible sources, either in school settings, clinical or community settings with or without parents present, or delivered by peers and which could moderate risk-taking among youth is critical. Such education should be clear that while HIV is treatable and that treatment can prevent its spread to others, nonetheless, having HIV doesn’t make one’s life any easier and its consequences are both physical and social. In this way, increasing stigma around people with HIV is avoided.”**

2) The authors state that increased awareness of ARVs and reduces HIV stigma, young people also express less concern about getting infected with HIV. Prior research shows that perceptions of ARVs as efficacious and readily accessible can be linked to lower levels of concern of HIV infection. It is crucial to consider the extent to which ARVs have transformed perceptions of HIV from a life-threatening condition to a manageable, long term, chronic condition – and how this may have resulted in disinhibition or risk compensation behaviour.

**We thank the reviewer for the insights. Not only have we added the above-mentioned new paragraph to the discussion, we also added a note in the introduction – "and crucially, the introduction of novel therapies or more effective medication" – to highlight ways that risk perception is modifiable. We did not, however, cite Legemate’s article, as the methods section there stated the researchers had disregarded all papers involving PrEP, which is the focus of our paper.**

3) I like the author’s use of scripting theories – a useful way to look at young men’s social competition, masculinities and gendered scripts. Other research has also shown that sexual prowess, proof of power over partners, and the prestige associated with condomless sex, and impregnating a female partner were are woven into gendered scripts for young men in sub-Saharan Africa. Young men prioritise prestige amongst peers, and demonstrations of their sexual maturity – at times proven by impregnating a girl. Condomless sex has also been associated with concepts of manhood and masculinity – regarded as more immediate and important than considerations around HIV infection.

**We thank the reviewer for this observation, and we recognise that the literature does contain this evidence, which was absent in our cohort. In our FGDs, however, our participants did not indicate an association between impregnating women and their sexual prowess; perhaps our demographic was too young – we believe that in our region, such attitudes might be more evident amongst married couples. Similarly, we did not find evidence of a discourse around condomless sex and masculine ideals; instead, we found saw that condomless sex is valued for increased pleasure (or the reverse: that condoms are eschewed because they decrease pleasure or have negative health-related side effects). We summarise these findings succinctly in the discussion as follows: “Intimacy in the sense of pleasure was prioritized (sweet in its pack), but in contrast to much earlier findings from other regions, we did not find evidence of foregoing the use of condoms in order to demonstrate either virility or intimacy in the sense of trust or commitment in this sample [40-43].”**

4) Another critical theme that this paper addresses relates to SRH communication gaps between adolescents and parents. Evidence shows that where there are barriers to effective SRH communication between parents and adolescents, girls end up getting advice from peers and making poorly informed decisions. But it is not always the fault of parents – girls also may not want to listen to parents, regarding them as outdated and irrelevant – highlighting the generation gap.

**We thank the reviewer for this great observation. We have reframed the requisite quote in the results section to draw this out. We have also adjusted the paragraph in the discussion to read as follows (including new citations you recommended): “Similar to other studies on the continent, youth also accused parents of failing to offer appropriate guidance, not only abandoning informational support to other sources like school teachers, but also offering inadequate material support to the young woman, leading her to seek support elsewhere [52]. A Botswana study also found parents’ misperceptions about their adolescent children’s relationships to be associated with higher risk activities on the part of the adolescents [53]. Although nothing guarantees that youth will listen to their parents, the accusation that they do not provide any guidance at all highlights the possibility that parents don’t have a ‘script’ of their own to provide their children. They can provide neither behaviours to illustrate, nor health beliefs to pass on – perhaps trusting that teachers teach the necessary information. This is similar to the pre-intervention state in at least one intervention study conducted in South Africa, which demonstrated success in overcoming barriers on both the youth and parental sides of the equation [52].”**

5) It would be beneficial if the authors also consider the complexity around transactional sex and relationships. Is it as straightforward as poverty being the sole driving motive for transactional relationships? What about gender and peer norms – around fashion, clothing, material displays of wealth and ‘glamour’ in an effort to gain peer acceptance and social status? How about the agency of young women in choosing to use their sexuality as a currency? The authors suggest that there is a lack of autonomy when young women engage in transactional sex. However evidence suggests that young women may also be enacting some level of sexual agency and using their attractiveness and sexual ‘value’ to attain things they want – i.e. education, upward social mobility?

**We are grateful for the observation. We would like to clarify that transactional sex is nuanced and composed of its own peer norms and agentic behaviour. We specify women do seek men who can pay for school fees; however, in the context of the interpersonal dynamics, women's sexual agency can be limited. We have greatly expanded this paragraph in the discussion, and the relevant portion now reads as follows: “Young women, in particular, expressed little control over partner behaviors or felt pressured to engage in transactional sex. This perception has some epidemiological foundation [44, 45]. Similar experiences among young adults in Malawi [30], Tanzania [46], and South Africa [45] underscore the widespread lack of autonomy young women have when engaging in transactional sex even when they initiate such relationships [47]. In the context of widespread transactional sex, youth believe older men contribute to HIV transmission, creating the perceived dynamic of increased infection rates among both young women and young men due to such age-asymmetrical relationships [45]. Transactional sex, however, is nuanced and has its own peer norms and agentic behaviour, and both women and men do sometimes seek older partners who can pay for school fees or buy them nice things in order to be competitive with their peers; however, in the context of the interpersonal dynamics, women's sexual agency in particular can be limited inasmuch as their reception of these material benefits is dependent on particular forms of sexual compliance [45, 46]. Further, this phenomenon produces different perceptions among men and women about each gender’s goals in pursuing sexual relationships, with men believing women are materialistic and women believing that men seek sex and will exchange material goods to obtain it. Together, the combination produces a feedback loop which raises the risk of HIV for everyone involved in this complex sexual economy. Ultimately, motivations to engage in transactional sex in our sample, as well as those from elsewhere on the continent, are driven by multiple, interpenetrating factors.”**

**6)** The authors state that for some young people, unprotected sex is excused as a norm that falls under the umbrella of enjoying their youth. Where does the prioritisation of pleasure play into this? Also, to what extent do young women prioritise relationship security over sexual health? Research amongst young people has shown that key factors determining condom use motivations for adolescent girls relate to relationship security and the desire to demonstrate love, trust, intimacy and commitment. In this manner, young women demonstrate a prioritization of romantic security and intimacy over their own sexual health, and seem to be willing to put themselves at risk for the sake of demonstrating trust, and prioritizing romantic connection. This can also be linked back to gendered scripting – and the framing of the importance of being in a relationship for young women = and the social prestige that brings. It has been shown that the self-esteem and social status of young women is often linked to their being in a romantic relationship, and therefore the security and maintenance of relationships is prioritized, which therefore can compromise risk avoidance behaviours.

**We appreciate the useful comments. Although we would like to link decision not to use condoms to gendered scripts, we did not see women saying they opted to forgo condoms in order to demonstrate trust; that was not in our dataset. However, relationship security was present -- hence the women's acquiescence when men wanted to not use condoms. Men did specify the priority of pleasure (condoms = eating sweets or candy still wrapped in its package). We have added the following sentences: "Intimacy in the sense of pleasure was prioritized (sweet in its pack), but in contrast to much earlier findings from other regions, we did not find evidence of foregoing the use of condoms in order to demonstrate intimacy in the sense of trust or commitment in this sample," and, "In our sample we did not find many young women who seemed to prioritise being in a steady relationship, so we cannot say that they prioritized (non-financially-motivated) relationship security over using condoms. This is perhaps an artefact of the age group of our sample, and that once older, more stable relationships better fulfill gendered expectations and scripts."**

7) Whilst gendered scripts are a critical consideration, it is also crucial to consider the way in which gendered power norms can also undergo shifts, and the ways in which young people can challenge them. Sexual scripts relating to gendered power are embedded in and informed by socio-cultural norms – which are themselves not static and unchanging – but are indeed slow to shift. Young people in sub-Saharan Africa navigate the duality between stubbornly persistent traditional values and expectations which co exist alongside more modern expectations.

**We thank the reviewer for the observation and have added the following to the limitations section: "Additionally, our focus in this paper has been on gendered social scripts, but gendered power norms, including within sexual relationships also undergo change. That topic has been explored among Ugandan adults in the early years of the HIV epidemic, but how youth receive these norms in the context of Test and Treat has been understudied"**

**Finally, we thank this reviewer for the list of references. While we did not incorporate all of them into the article, those we did cite and discuss have improved the quality of our paper. We also believe that other team members, who did not contribute to this particular article, but whose research interests align with the suggested references, will also find those articles useful for their future publications. We are accordingly sharing the list with the rest of the team(s).**

*Reviewer 2 Comments to the Author*

Well-written paper that adds to the literature around HIV risk perception among young people. The rules guiding the HBM in the scientific study were delicately respected. The quotes are informative and lend valuable insights. A few thoughts to improve the paper.

**We appreciate the reviewers’ thoughtful comments and encouragement.**

Introduction

Line 36: Is there any work from the parent study that was published? It will be good to give the citation here.

**We appreciate the reviewers’ observation and have added the appropriate citations to both the introduction and methods sections. (References 14, 15, 16, and 34)**

Line 39-40: Some of these details fit best in the method section. Please clarify if this qualitative study was nested in a larger study given the details provided here.

**We agree with the reviewer that some of these details would fit well in the methods section; we've provided them here because of how they relate to our argument about contextual factors which change risk perception, and felt the introduction was the best place to do that. (For context, the relevant section reads: “Many people learned their HIV status for the first time [during this trial], which led to increased disclosure and consequent fear of stigma from disclosure, but also magnified linkage to care. Further, PrEP was offered at the end of Phase 1, which aimed at using streamlined care delivery to measure the impact of early diagnosis and ART delivery. *Any one of these rapid changes could affect youth HIV risk perception. Understanding HIV risk perceptions among youth in SSA requires consideration of this dynamic character*.”)

We have also clarified in the opening sentence of the methods section that the qualitative study was nested within this larger study: "We use data collected through a qualitative study that was nested in the larger study Sustainable East Africa Research in Community Health (SEARCH) trial, a population-based HIV test-and-treat trial implemented in Ugandan and Kenyan communities…"**

Line 64: After introducing the study, the author goes ahead to describe how other people have used the theory which I think may not be necessary unless the paper aims to improve the tenets of the theory, which is not the case here since the theory is only used to better understand the findings. A brief description of the theory and its application would suffice right before introducing your study. This also helps in being concise and focusing the reader on your work. Other details may be included in the discussion where appropriate.

**We appreciate the reviewer's kind suggestion for how to make the paper more concise. However, we are indeed trying to improve the tenets of the theory by incorporating social scripts as a specific but overlooked modifying variable as described in the paragraph that follows this one. We have therefore opted to keep this paragraph intact -- but thank the reviewer for the suggestion.**

Methods

Line 130: Consider describing the high HIV prevalence in the two regions and provide relevant citations.

**We thank the reviewer for the helpful observations. We now specify “a population-based HIV test-and-treat trial implemented in Ugandan and Kenyan communities with adult HIV prevalence from 7.9% (SW Uganda) to between 14.3% and 26% (Migori, Kisumu, and Homa Bay counties, Kenya) [31-33].” – thereby also including new citations.**

Line 132: Not sure about ‘boda boda’ drivers being a globally universal word.

**We appreciate the reviewers’ observation and have clarified further that bodaboda drivers “motorcycle drivers”.**

Line 134: Write FGD in full for the first mention.

**We thank the reviewer for catching this. We have expanded the first mention of FGD in full as “Focus Group Discussion”**

Line 135: Using the word ‘we decided to concentrate on’ may not be very appropriate here. Instead, just mention what the study did e.g. The study used FGDs…and give the rationale for using the FGDs.

**We thank the reviewer for this suggestion, and ordinarily agree that this is the best approach. However, we specified our focus because a previous reviewer castigated us for only using the FGDs rather than both FGDs and IDIs. Nevertheless, we have shorted the phrasing to "we focused on FGDs alone because..."

Additionally, because of the later concern around six researchers coding, we wanted to be clear that the team coded both IDIs and FGDs together -- hence the larger team effort.**

Line 143: Please clarify if community resident youth are the study participants it is not very clear. This statement ‘the LAM represented’… ‘they were further balanced’…I suppose these were mobilizers, right? So FGD participants were purposively sampled based on?

**We appreciate the observation and have changed the sentence to read as follows “Community resident youth who participated in the FGD were purposively sampled for age, gender and having lived in the community for the past 6 months, through SEARCH-recruited Local Area Mobilizers (LAM) drawn from different social groups”.**

Line 150: What is the rationale for conducting an FGD with up to 17 participants in one sitting? given the threshold is 6-12, and given group dynamics? Why was this FGD not divided into two?

**We like that the reviewer noticed this, since it gives us the opportunity to clarify that the reason is due to cultural practices in that particular region of Uganda. (Our Ugandan teammates clarified that to us when we inquired based on this concern.) We note this now by stating: "one larger FGD in Southwestern Uganda (where cultural norms dictate accompanying one another to social forums, leading to larger gatherings), and two in Kenya ..."**

How were the FGDs conducted? i.e. how many moderators and note-takers please clarify.

**We respect the kind concerns by the reviewer, which also align with COREQ guidelines, and have added “Each FGD had one moderator and one field-note taker.” to clarify further.**

Line 152: Consider the number of FGDs per site and how they were distributed or refer to (table 1).

**We appreciate the reviewers’ concern and have enriched the title as follows “*Focus Group Distribution by Community and Participant Numbers”.***

Line 154: Consider giving a brief description of your FGD guide, and what it elicited.

**We appreciate the reviewers’ insights and have added a sentence as follows to address the concern: "The FGD guides assessed attitudes, beliefs, social group norms related to PrEP as well. Further, it helped in gaining target population insights into potential implementation strategies".**

Line 158: Name table 1.

**We thank the reviewer for the observation. We have named the table as “*Focus Group Distribution by Community and Participant Numbers”***

Line 161: 6 researchers? What role did each researcher play in the analysis? The author’s reporting does not adhere to the COREQ guidelines for reporting qualitative work. Check that your methods align with this in your methods.

**We consider this concern and respectfully changed the statement to: “We developed a coding framework after reviewing a subset of transcripts where all coders were assigned two similar transcripts for inductive coding.”**

Were there any intercoder agreements to ensure there was consensus in the coding process, please describe.

**We thank the reviewer for the concern. We have added the following sentence “Whilst coding, the team met regularly to review any questions. Group members were assigned both IDIs and FGDs to code using the same codebook.”**

Provide citation or web-based link for dedoose software.

**We appreciate the suggestion and have added the relevant citation (36).**

Line 176: NASCOP, KEMRI, and UNCST say ≥ 12 can consent, please clarify under what circumstances and provide relevant citations.

**We appreciate the reviewer’s question. We clarify the statement by replacing “such research” with “research about STIs, including HIV”.**

Also, for clarity, indicate that participants provided written informed consent.

**We appreciate the reviewers’ suggestion and made the following addition “Youth in our study provided independent, written consent for their participation”.**

Results

Were there any demographic data collected from the FGD participants? e.g. social demographic information?

**We thank the reviewer for the opportunity to clarify. We did not collect social demographic information beyond ethnicity and age, as all participants were school-age and school-going. We therefore do not report exact breakdowns in these relatively ethnically homogeneous rural communities.**

Line 183: Please provide a general summary overview of your findings before expanding the themes.

**We agree such a frame is needed, and have adjusted the initial paragraph in this section as follows: “****Tightly connected social networks and widespread uptake of antiretrovirals shaped youth HIV risk perception. We found complex and varied risk perceptions among participants showing that their health beliefs were influenced by their social ties in each aspect we studied. Below we decompose the category of *perceived threat* into perceptions of risk and perceptions of severity, before we describe the resulting interaction of perceived threat with youth-reported observations about *peer norms* as they influence the uptake of PrEP and other behaviours. Gendered differences in the dynamics of risk susceptibility were evident, while perceptions of the severity of HIV showed less distinct variations by gender, as noted below. We specify participants by their FGD participant number (e.g., “P5”), sex, community, and country.”**

Line 161: You describe 4 tenets in the HBM but only describe 2 in the findings please clarify.

**We thank the reviewer for the opportunity to clarify. In the background section we state, "This paper, informed by critiques of the HBM, differs from previous work by exploring how modifying factors influence HIV risk perceptions." We earlier noted that, "Therefore, the most useful parts of the HBM model to focus on concern perceptions that are amenable to peer norms." -- this latter instance was italicised precisely to point out why we describe only two tenets in the findings.**

Discussion

Line 571: Please clarify did you explore the constructs of HBM or did you explore HIV risk mechanisms framed in the context of HBM?

**We appreciate the good catch and have now rephrased the sentence to read: "In this qualitative study conducted in the context of the SEARCH test and treat trial during the widespread offering of PrEP, we sought to explore the mechanisms of risk perception as framed in the construct of modifying factors from the Health Belief Model among young men and women ages 15 to 24 in rural Kenyan and Ugandan communities."**

References

Some references are rather old e.g. 1991, 2000 Please confirm that there is no recent literature for this work.

**While we agree that it is important to use the most recent literature possible, these references were cited in a paragraph specifically concerned with the relevant history of how the HBM model has been used on the continent: “The HBM has been applied to understanding risky behavior among youth in sub-Saharan African settings with varying results since the early 1990s [18]. However, the utility of HBM for HIV prevention behaviour could not be confirmed for condom use among students in late 1990s / early 2000s in South Africa [19]. Later, researchers there found that HBM predicts only about 25% of the acceptability of routine HIV testing among students, with *perceived benefits* having the greatest weight [20]. In contrast, Oyekale and Oyekale used the HBM to actively promote behaviour change among Nigerian youths in 2010.”**

Consider providing a web-based link for citations 6,7,8.

**We have added these and hope that our reference software, where these URLs are stored, does not eliminate our manual adding of them to the reference section of the paper!**

Please review your citations, and be sure to use the right referencing tool for uniformity.

**We appreciate the concern by the reviewer. We have reviewed the citations and corrected any which listed ‘Organization’ as a surname (or similar mis-citations) within our referencing software for future consistency.**

**Again, we appreciate the close, thorough, and helpful comments provided by the reviewers and editor. Thank you for the opportunity to have our manuscript considered for publication in PLOS Global Public Health.**

**Sincerely,**

**Lawrence Owino and Jason Johnson-Peretz**
